# Reparative effects of interleukin-1 receptor antagonist in young and aged/co-morbid rodents after cerebral ischemia

**DOI:** 10.1016/j.bbi.2016.11.013

**Published:** 2017-03

**Authors:** Jesus M. Pradillo, Katie N. Murray, Graham A. Coutts, Ana Moraga, Fernando Oroz-Gonjar, Herve Boutin, Maria A. Moro, Ignacio Lizasoain, Nancy J. Rothwell, Stuart M. Allan

**Affiliations:** a Faculty of Biology, Medicine and Health, University of Manchester, Manchester, UK; b Unidad de Investigación Neurovascular, Departamento de Farmacología, Facultad de Medicina, Universidad Complutense (UCM) and Instituto de Investigación 12 de Octubre (i+12), Madrid, Spain; c Department of Neurology, Yale School of Medicine, New Haven, USA

**Keywords:** Cerebral ischemia, Interleukin-1 receptor antagonist, Neuroprotection, Neurogenesis, Co-morbidity, Risk factors

## Abstract

Neuroprotective strategies for ischemic stroke have failed to translate from bench to bedside, possibly due to the lack of consideration of key clinical co-morbidities. Stroke and co-morbidities are associated with raised levels of the pro-inflammatory cytokine interleukin-1 (IL-1). Inhibition of IL-1 by the administration of interleukin-1 receptor antagonist (IL-1Ra) has shown to be neuroprotective after experimental cerebral ischemia. Stroke can also trigger a robust neuroreparative response following injury, yet many of these new born neurons fail to survive or integrate into pre-existing circuits. Thus, we explore here effects of IL-1Ra on post-stroke neurogenesis in young and aged/co-morbid rats. Aged lean, aged Corpulent (a model of atherosclerosis, obesity and insulin resistance) and young Wistar male rats were exposed to transient cerebral ischemia, received subcutaneous IL-1Ra 3 and 6 h during reperfusion, and effects on stroke outcome and neurogenesis were analyzed. Our results show that administration of IL-1Ra improves stroke outcome in both young and aged/co-morbid rats. Furthermore, IL-1Ra not only increases stem cell proliferation, but also significantly enhances neuroblast migration and the number of newly born neurons after cerebral ischemia. Overall, our data demonstrate that systemic administration of IL-1Ra improves outcome and promotes neurogenesis after experimental stroke, further highlighting the therapeutic potential of this clinically approved drug.

## Highlights

• IL-1β is a key proinflammatory cytokine involved in ischemic brain damage.
• Administration of IL-1Ra improves the stroke outcome in young and co-morbid rats.
• Acute IL-1Ra administration also promotes neurogenesis after experimental stroke.

## 1 Introduction

Cerebral ischemia is the second leading cause of death and disability worldwide with treatment limited to fibrinolysis and intravascular therapy, both applicable in a low percentage of patients. Other strategies to improve outcome after stroke have failed to translate from the preclinical setting to the clinic, and with these failures have come calls for more rigorous and transparent approaches to experimental design and conduct (Albers et al., 2011, Howells et al., 2014). Highlighted in many of these calls is the need to consider age and co-morbidities experimentally, as these involve a strong inflammatory response, and are associated with increased risk of stroke and poor post-stroke outcomes.

The pro-inflammatory cytokine interleukin-1 (IL-1) is a major driver of inflammation, with well documented detrimental effects in multiple preclinical models of systemic inflammatory disease as well as in cerebral ischemia (Denes et al., 2010, Fan et al., 2013, McColl et al., 2007). To this end, the selective, naturally occurring competitive inhibitor of IL-1, interleukin-1 receptor antagonist (IL-1Ra) has shown potential as a new treatment for stroke (Emsley et al., 2005, Banwell et al., 2009, Smith et al., 2012). More specifically, in a number of experimental stroke paradigms IL-1Ra reduces infarct volume and improves long term functional outcome, including in co-morbid animals (McColl et al., 2007, Relton and Rothwell, 1992, Pradillo et al., 2012, Girard et al., 2014). However, exact mechanisms by which IL-1Ra is neuroprotective are yet to be fully established.

While much research has focused on limiting ischemic damage in the initial stages of acute reperfusion, it is also important to understand mechanisms that underpin brain repair following injury and develop strategies that enhance reparative endogenous processes, including adult neurogenesis. Ischemic injury elicits a robust neurogenic response (Arvidsson et al., 2002) by stimulating production of neuronal progenitor cells (NPCs) in distinct neurogenic regions, which include the subventricular zone (SVZ) and the subgranular zone (SGZ), to generate new functional neurons. Though mechanisms underlying post-stroke neurogenesis and the influence of inflammation on these processes are still poorly understood, it has been observed in young and aged animals that inflammation impairs both basal levels of neurogenesis and attenuates the neurogenic response triggered by CNS injury via induction of the pro-inflammatory cytokines (Vallieres et al., 2002, Ben-Hur et al., 2003, Wong et al., 2004, Bachstetter et al., 2011). IL-1, for example, reduces the proliferation and differentiation of NPCs to neurons in pathologies such as stress and depression, effects reversed by administration of IL-1Ra (Koo and Duman, 2008, Zhang et al., 2013).

Here, we explored how inhibition of IL-1 actions by clinically relevant, delayed administration of subcutaneous IL-1Ra affects stroke outcome and neurogenesis up to 28 days after experimental ischemia, in aged/co-morbid and young rats.

## 2 Materials and methods

### 2.1 Animals

All experiments were performed using 13-month-old male, lean (JCR:LA-lean (cp/); 400–500 g) and Corpulent (Cp) (JCR:LA-cp (cp/cp); 900–1000 g) rats (University of Alberta, Edmonton, Canada) and 2-month-old Wistar rats (200–250 g; Charles River, Wilmington, MA, USA). Cp rats are homozygous for the autosomal recessive cp gene (cp/cp), and spontaneously develop obesity, hyperlipidemia, insulin resistance, glomerular sclerosis, and atherosclerosis with enhanced vascular contractility and reduced vascular relaxation (Mangat et al., 2007). Animals were allowed free access to food and water and were maintained under temperature, humidity, and light-controlled conditions. All procedures were performed under appropriate United Kingdom Home Office licenses and adhered to the Animals (Scientific Procedures) Act (1986) Kilkenny et al., 2010.

### 2.2 Treatment

Animals were randomized for all experiments and assessments were performed in a blinded manner. Lean, Cp and young Wistar rats received two doses (subcutaneous) of placebo or IL-1Ra (25 mg/kg, 12.5 mg/kg and 50 mg/kg respectively) at 3 and 6 h post reperfusion and were allocated randomly to the following experimental groups sacrificed on post-stroke day 7: aged lean + tMCAO + placebo (*n* = 10); aged lean + tMCAO + IL-1Ra (n = 10); aged Cp + tMCAO + placebo (n = 9); aged Cp + tMCAO + IL-1Ra (n = 9); young Wistar + tMCAo + placebo (n = 8) and young Wistar + tMCAo + IL-1Ra (n = 8). Another two groups of young Wistar were sacrificed at 14d after stroke: young Wistar + tMCAo + placebo-14d (n = 8) and young Wistar + tMCAo + IL-1Ra-14d (n = 8) to determine neuroblast migration, and two groups at 28d: young Wistar + tMCAo + placebo-28d (n = 8) and young Wistar + tMCAo + IL-1Ra-28d (n = 8) to analyze the number of new integrated neurons. Group sizes were determined by power calculation (α = 0.05, β = 0.2).

The pharmacokinetic profile of subcutaneous human IL-1Ra (r-met-huIL-1Ra: Kineret; Amgen, Thousand Oaks, CA, USA) or placebo (Amgen, Thousand Oaks, CA, USA) was assessed in young and aged-comorbid rats as previously described (Greenhalgh et al., 2010). Owing to the highly lipophobic formulation of the drugs, obese Cp rats received half the dose of IL-1Ra (50%) per body weight compared with aged lean rats (Pradillo et al., 2012). Plasma levels of IL-1Ra (measured by ELISA) in Lean/Cp rats reached a concentration of ∼8000 ng/ml 8 h after both injections. Young Wistar rats were given double the dose of the aged lean animals due to the faster metabolism of young animals (Mondon and Starnes, 1992). Following studies by Greenhalgh et al. (2010), a single administration of 100 mg/kg of IL-1Ra to young rats at the time of the MCAO, resulted in a plasma concentration of ∼9000 ng/ml 8 h after its administration. Therefore the administration regime of IL-1Ra used here resulted in comparable plasma levels of drug, at what we believe to be clinically therapeutic concentrations (Emsley et al., 2005).

### 2.3 Focal cerebral ischemia

Focal cerebral ischemia was induced in aged lean and Cp rats by 90 min transient occlusion of the left middle cerebral artery (tMCAO) as described previously (Pradillo et al., 2012). Focal cerebral ischemia was induced in young Wistar rats by 70 min transient occlusion of the left middle cerebral artery and left common carotid artery (CCA). The slight difference in surgical protocol was due to a resistance of young rats to infarction when only the MCA was occluded (data not shown). It was necessary to occlude both the MCA and CCA in these young healthy animals to achieve similar infarcts to aged lean/corpulent rats infarcts to analyze the IL-1Ra effect on neurogenesis, due to the influence of infarct size in the neurogenic response after stroke (Moraga et al., 2014). Isoflurane (2% for induction and 1.5% during surgery) was used in a mixture of 70% N_2_O and 30% O_2_. Core body temperature was maintained at 37.0 °C ± 0.5 °C throughout the surgery by a heating blanket (Homeothermic Blanket Control Unit; Harvard Apparatus, Kent, UK). Ischemia was induced by a transient ligature of the left MCA trunk and/or CCA with a 10–0 suture (Prolene, Ethicon, Somerville, NJ, USA). Occlusion and reperfusion were confirmed visually under the surgical microscope. After surgery, animals were returned to home cages and allowed free access to water and food. Animals were excluded from statistical analysis based on an a priori exclusion criterion, namely if animals experienced brain hemorrhage, lack of reperfusion or if the surgery took longer than 45 min and there was excessive bleeding (2 aged leans were excluded in total). Only one animal died during the duration of the study due to anesthetic overdose.

### 2.4 Measurement of infarct volume and BBB damage

Lesion volume and edema were assessed on T_2_W brain images obtained at 24 h and 7 d after stroke, using a 7-T, horizontal-bore magnet (Agilent Technologies, UK) interfaced to a Bruker Avance III console (Bruker Biospin, UK) using a surface transmit-receive coil. Images were analyzed using Anatomist software (http://brainvisa.info). In the group of young Wistar rats sacrificed at 28 d, the loss of cortex was determined in brain sections by Nissl staining as described previously (Pradillo et al., 2012).

Blood–brain barrier (BBB) damage was determined by immunohistochemical staining of endogenous rat immunoglobulin G (IgG) as described previously (Greenhalgh et al., 2010) in all the experimental groups at 7d, and in young Wistar rats at 14d of reperfusion.

### 2.5 Stroke outcome

Neurological status was assessed blinded to drug treatment, before and at different time points up to 28d after stroke, and by the use of motor, behavioral and cylinder tests (Hunter et al., 2000, Madrigal et al., 2003, Schallert et al., 2000). For the motor and behavioral scales, each test was conducted three times per trial and the average taken to determine outcome. For the cylinder test, each animal was allowed to freely explore the arena for up to 4 min. All rears were scored by the use of the right paw, left paw, or both paws during rearing (Encarnacion et al., 2011). The different forelimb preference, for placements made, was calculated as a percentage of the total number of placements made.

### 2.6 Incorporation of bromodeoxyuridine (BrdU)

To examine the number of new integrated neurons in young Wistar rats at 28 d after experimental stroke, animals were injected intraperitoneally with 50 mg/kg bromodeoxyuridine (BrdU; Sigma; UK) twice a day on days 4, 5 and 6 post-ischemia.

### 2.7 Tissue processing

Rats were transcardially perfused with 0.9% saline followed by 4% paraformaldehyde (pH = 7.4). Brains were removed, post fixed, cryoprotected in 30% sucrose and frozen at −20 °C. Sections (30 μm) were cut on a sledge microtome (Bright series 8000; Bright Instruments, Huntingdon, UK) and stored at −20 °C in antifreeze solution (30% ethylene glycol and 20% glycerol in phosphate-buffered saline) until processing.

### 2.8 Immunofluorescence

With this technique we evaluated different markers of neurogenesis. We first analyzed NPCs proliferation in the SVZ by staining for Ki67, a nuclear protein associated with cellular proliferation which is expressed in all phases of the cell cycle, except the resting phase (Kee et al., 2002). To determine the differentiation of NPCs into neuroblasts, we stained for Doublecortin (DCX), a microtubule associated phosphoprotein which is a neuroblast marker (Rao and Shetty, 2004). Finally, we studied the number of mature differentiated and integrated neurons (NeuN^+^/BrdU^+^/cFos^+^ cells) as described previously (Kee et al., 2007), with slight modifications. For these purposes free-floating sections (30 μm thick) were washed in PBS and incubated for 120 min in blocking solution (PBS, 0.5% Triton X-100 and containing 5% donkey serum, Serotec, UK) and then incubated overnight at 4 °C in blocking solution with the following primary antibodies (Ab): rabbit anti Ki-67 (Abcam, UK); goat anti Doublecortin (Santa Cruz Biotechnology, USA); mouse anti NeuN (Millipore, UK) and rabbit anti c-Fos (Abcam, UK). Antigens were visualized by using the following fluorochrome-conjugated secondary Abs: donkey-anti rabbit, anti-goat or anti-mouse Alexa Fluor 488 to detect Ki-67, DCX or NeuN, respectively, and donkey-anti rabbit Alexa Fluor 647 to detect c-Fos (Invitrogen, UK). Sections used to detect NeuN and c-Fos were then fixed for 15 min in 4% paraformaldheyde solution, washed with PBS and then the BrdU detection was started. Sections were first incubated in HCl 2 N at 37 °C for 30 min in order to denature the DNA chain and, after washing in PBS, were incubated in blocking solution as described above. BrdU antigen was visualized by using a sheep-anti-BrdU Ab (Abcam, UK) and an anti-sheep Cy3 as secondary Ab (Millipore, UK). Finally, all sections were washed with PBS and mounted onto slides and cover slipped with Prolong anti-fade medium (Life Sciences, Paisley, UK). Controls performed in parallel without primary antibodies showed very low levels of nonspecific staining.

### 2.9 Optical density and cell quantification on confocal images

All images were acquired in a blinded manner by laser-scanning confocal microscopy (LSM710; Zeiss, Munich, Germany). To analyze the proliferation of NPCs labeled by Ki67, four images from five consecutive sections were captured to cover the total extension of the SVZ per animal, starting at 1.70 mm from bregma until -0.40 mm (Supp. Fig. 4A; regions A to D). The distance between image A and B was 200 μm; distance between B-C and C-D was 400 μm, and z-stacks of each image were obtained at 20x.

To study the effect of IL-1Ra on neuroblasts migration (DCX^+^ cells) we follow the protocol described previously by Moraga et al., in 2014, where the migration of these cells was studied in mice after stroke (Moraga et al., 2014). Then, immunofluorescence z-stack images were obtained at 20× from five correlative sections of each brain. We established three different zones to analyze the neuroblast migration, starting at the SVZ (zone 1) and two adjacent zones (Zone 2 and Zone 3) along the Corpus Callosum (see Fig. 5A). The analysis of both, the effect of IL-1Ra on Ki67 immunostaining and DCX^+^ cells migration images were performed by integrated density calculations using Volocity 3D image analysis software (Perkin-Elmer, USA).

For the analysis of the number of new integrated neurons (BrdU^+^/NeuN^+^/c-Fos^+^ cells), immunofluorescence images were taken from 4 consecutive sections beginning at 1.60 mm from bregma until 0.20 mm. The images were taken at 40x, spaced 800 μm from each other to cover the entire top of the cortex below the stroke, using as boundaries the corpus callosum and the end of the cortex (see Fig. 6A and Supp. Fig. 4B). A total of 30 images/ipsilateral- hemisphere/section were obtained, and the quantification of the new neurons was made using ZEN 2009 software (Zeiss). All colocalization images shown were confirmed by orthogonal projection of the z-stack files.

### 2.10 Statistical analysis

Group sizes (n = 6–10) were calculated based on previous data. Data are presented as mean ± standard deviation (SD). For parametric data, Student’s *t*-test and two-way ANOVA followed by Bonferroni’s correction were used for single and multiple comparisons respectively. For non-parametric data, Mann-Whitney *U* test followed by Bonferroni’s correction was performed. Non-parametric data are presented as median distribution (interquartile range). Linear association between 2 variables was determined by the Pearson correlation coefficient.

## 3 Results

### 3.1 IL-1Ra improves long-term stroke outcome

**Fig. 1:**
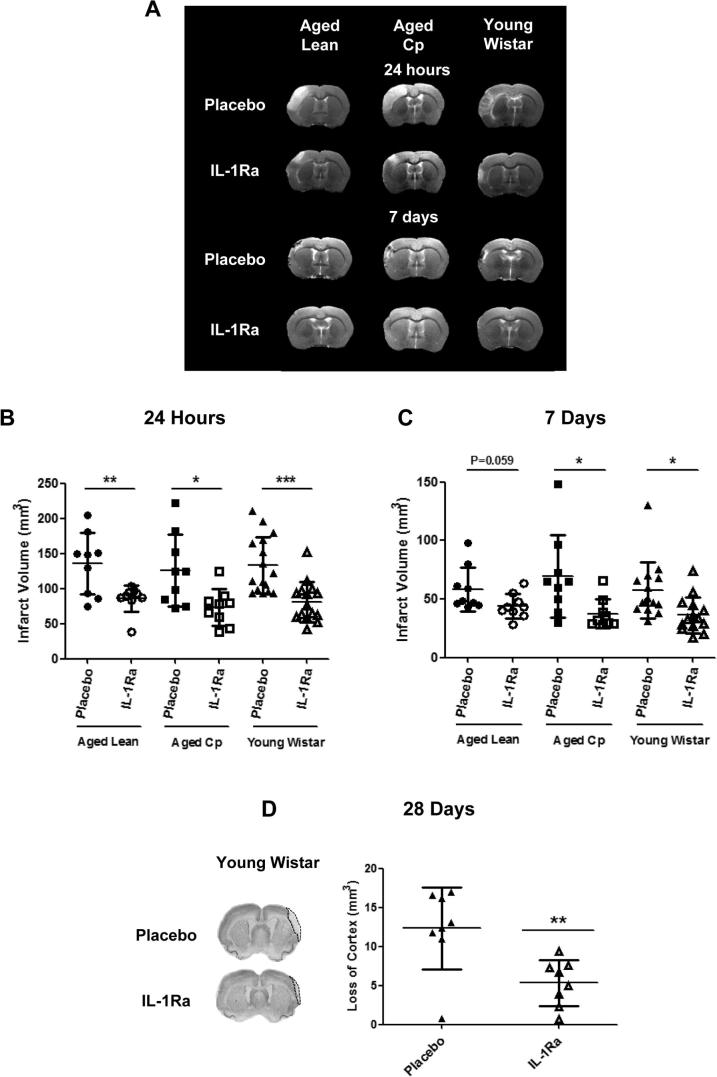
Effect of delayed administration of IL-1Ra/placebo (3 and 6 h of reperfusion) on brain injury. **A**: representative images of brain lesions in the different experimental groups. Total infarct volume (mm^3^) measured at 24 h (**B**) and 7d (**C**) after tMCAo in T_2_W images in aged lean (n = 10), aged Cp (n = 9) and young Wistar rats (n = 8). **D**: loss of cortex measured by Nissl staining in young Wistar rats at 28d (n = 8). Data are expressed as mean ± SD. ^*^P < 0.05, ^**^P < 0.01, ^***^P < 0.001, Student’s *t*-test.

Delayed IL-1Ra administration at 3 and 6 h reperfusion in aged lean, aged Cp and young Wistar rats induced a significant reduction in infarct volume at 24 h and 7d of reperfusion, and a significant reduction in cortex loss at 28d in young Wistar rats. Reductions in infarct volume at 24 h of reperfusion were 37%, 42% and 40% in aged lean, aged Cp and young Wistar rats respectively as measured by T_2_W MRI (Fig. 1A and B). A reduction in ischemic damage in IL-1Ra treated animals was also seen at 7d reperfusion in aged lean, aged Cp and young rats (24%, 46% and 37% of reduction respectively), although only reached significance in aged Cp and young Wistar rats (Fig. 1C). A reduction in cortex loss of 56% was also observed by Nissl staining in the IL-1Ra treated young Wistar group at 28d reperfusion compared to the corresponding placebo treated group (Fig. 1D). Edema was also measured and corrected for at 24 h and 7d reperfusion in all the animals following ischemic insult and there was no difference between placebo and IL-1Ra in any experimental group (data not shown).

**Fig. 2:**
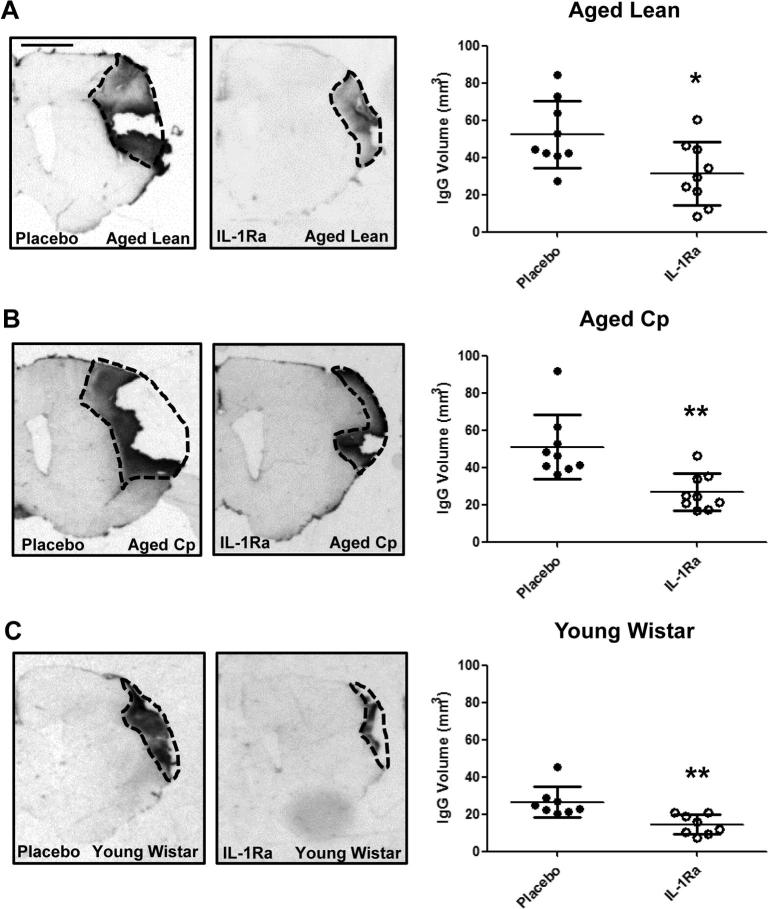
Effect of delayed administration of IL-1Ra/placebo on BBB damage measured at 7d after tMCAo in aged lean (**A**; n = 10), aged Cp (**B**; n = 9) and young Wistar (**C**; n = 8). The volume of BBB damage was calculated on brain sections after IgG staining. Data are expressed as mean ± SD. ^*^P < 0.05, ^**^P < 0.01, Student’s *t*-test. Scale bar: 2 mm.

IgG staining at 7d reperfusion revealed a reduction of 40%, 48% and 46% in BBB damage in IL-1Ra treated aged lean, aged Cp and young Wistar animals respectively, versus their placebo-treated counterparts (Fig. 2A–C). A reduction of 26% was also observed at 14d reperfusion in young Wistar rats treated with IL-1Ra versus their placebo counterparts (Supp. Fig. 1).

**Fig. 3:**
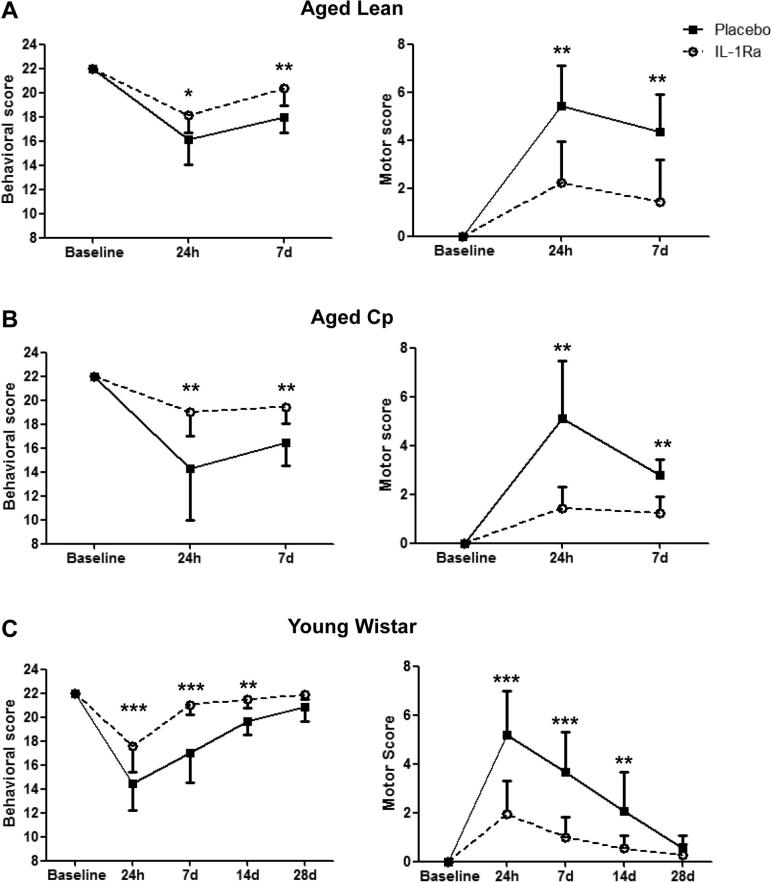
Effect of IL-1Ra on behavioral and motor deficits**.** Behavioral and motor scores were assessed before, 24 h and 7d after tMCAo in aged lean (**A**; n = 10), aged Cp (**B**; n = 9) and in young Wistar animals also at 14 and 28d after cerebral ischemia (**C**; n = 8). Data are presented as median and interquartile range. ^*^P < 0.05, ^**^P < 0.01, ^***^P < 0.001, Mann-Whitney *U* test with Bonferroni correction.

To assess the impact of delayed IL-1Ra on long term outcome, motor function and behavior were assessed by different scales and by the cylinder test, before and at 24 h and 7d following reperfusion in all animals, and in additional cohorts of young Wistar rats at 14d and 28d. Behavioral and motor scores showed all groups exhibited significant impairment at 24 h compared to baseline. Aged lean, aged Cp and young Wistar rats treated with IL-1Ra did however exhibit significantly better performance and smaller deficits at 24 h and 7d reperfusion versus their control counterparts (Fig. 3A–C). Placebo and IL-1Ra treated young Wistar rats returned to baseline by 28d reperfusion, though IL-1Ra treated animals had better outcomes versus placebo at 14d (Fig. 3C).

The cylinder test, used to measure forepaw asymmetry when the animal rears, revealed no bias of left and right forepaw usage in any of the groups prior to injury. At 24 h all animals (placebo or IL-1Ra) displayed deficits in the usage of impaired ipsilateral and contralateral forepaw, which began to recover by 7d post-tMCAo (Supp. Fig. 2A–C). IL-1Ra treated lean and young rats showed improvements in total number of rears versus their placebo treated counterparts at 24 h and 7d (Supp. Fig. 3A and C). There were no differences in any groups in the use of ipsilateral forepaw, contralateral forepaw or both forepaws (data not shown) at any time point examined (Supp. Fig. 2A–C).

### 3.2 IL-1Ra increases neurogenesis

**Fig. 4:**
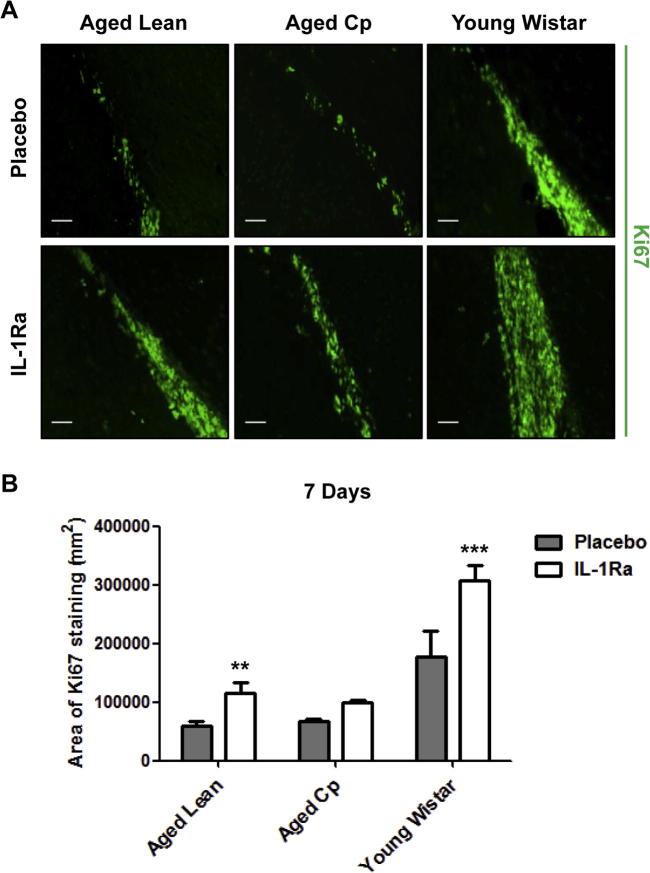
Effect of IL-1Ra on NPCs proliferation. **A**: Representative images of Ki67 immunostaining at 7d after tMCAo in aged lean, aged Cp and young Wistar (n = 6). **B**: Area of Ki67 immunostaining along the SVZ quantified in 5 consecutive brain sections/brain. Data are expressed as mean ± SD. ^*^P < 0.05, ^**^P < 0.01, ^***^P < 0.001, two-way ANOVA with Bonferroni correction. Scale bar: 50 μm.

Neurogenesis markers were examined by immunofluorescence at 7d reperfusion in aged leans, aged Cp and young Wistar rats, and only in the latter at 14d and 28d. We first analyzed proliferation of NPCs in the SVZ at 7d reperfusion using Ki67, a nuclear protein associated with cellular proliferation. We identified Ki67 immuno-positive cells in the SVZ, indicating the existence of NPCs proliferation in both placebo and IL-1Ra treated animals. An increase in the area of NPC proliferation was seen in all animals treated with IL-1Ra versus placebo-treated, though this was statistically significant only in aged lean and young Wistar rats (Fig. 4A and B). In agreement with existing literature (Moraga et al., 2014, Moraga et al., 2015), our results showed a positive but not significant correlation between lesion size at 24 h and proliferation of NPCs at 7d in young Wistar rats (R^2^ = 0.87, p = 0.06) and no correlation for aged-comorbid rats (i.e. aged Lean rats: R^2^ = 0.2, p = 0.44).

**Fig. 5:**
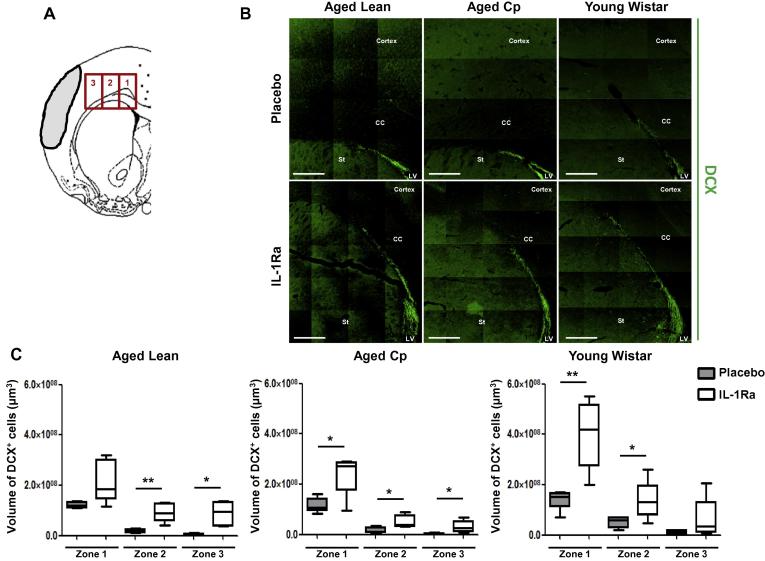
Effect of delayed administration of IL-1Ra on neuroblasts migration in aged lean, aged Cp and young Wistar rats at 7d after transient cerebral ischemia (n = 6). **A**: Brain template showing the three different zones analyzed to determine DCX^+^ cells migration; **B**: representative images of each experimental group; **C**: number of neuroblasts (DCX^+^ cells) in the 3 migratory zones at 7d after tMCAo in placebo or IL-1Ra treated animals rats. Data are expressed as mean ± SD. ^*^P < 0.05; ^**^P < 0.01, Student’s *t*-test. Scale bar: 400 μm.

To determine if NPCs have differentiated into immature neurons (neuroblasts) we stained for doublecortin (DCX), a microtubule associated phosphoprotein used as a neuroblast marker, and analyzed their migration through the corpus callosum towards the infarcted area. We identified DCX^+^ neuroblasts in the SVZ (zone 1) that migrated to the infarct (zones 2 and 3) in both placebo and IL-1Ra treated animals. When compared with placebo treated-animals IL-1Ra treatment increased the total number of immature neurons in the SVZ, as well as the migration of these cells to the infarct, in all groups at 7d of reperfusion (Fig. 5B and C), and in young animals at 14d (Supp. Fig. 5A and B).

**Fig. 6:**
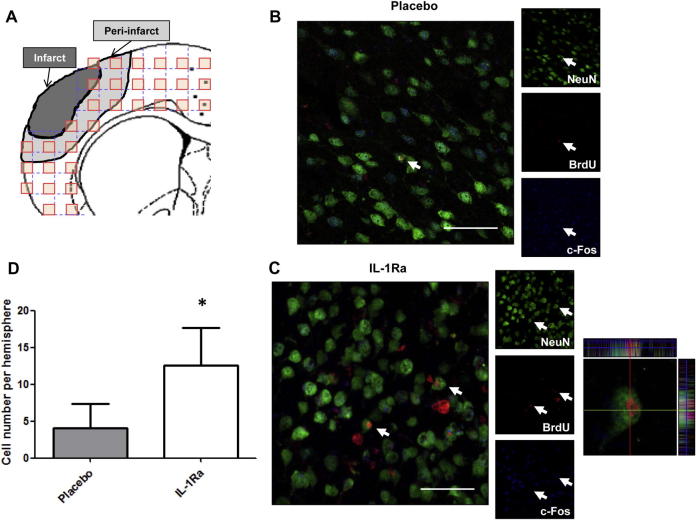
Effect of delayed administration of IL-1Ra on the number of new integrated neurons in young Wistar rats at 28d after transient cerebral ischemia (n = 6). **A**: Brain template showing the cortical regions of analysis, appearing in grey the region of the peri-infarct where the new neurons were found. **B** and **C**: Representative images of the different experimental groups, where the triple positive cells are shown by white arrows. **D**: Number of new integrated neurons (NeuN^+^/BrdU^+^/c-Fos^+^ cells) per hemisphere at 28d after tMCAo in placebo or IL-1Ra treated young Wistar rats (n = 6). Data are expressed as mean ± SD. ^*^P < 0.05, Student’s *t*-test. Scale bar: 25 μm.

We also studied the number of mature differentiated and integrated neurons (NeuN^+^/BrdU^+^/cFos^+^ cells) in the ipsilesional cortex of young Wistar rats at 28d after the ischemic insult. Delayed IL-1Ra treatment resulted in increased numbers of new integrated neurons compared to the placebo treated animals (Fig. 6B–D), demonstrating that IL-1Ra not only protects the ischemic brain but also increases neurogenesis after stroke.

Finally, we analyzed if there was any correlation between the improvement in functional outcomes seen in the IL-1Ra treated groups with markers of neurogenesis (NPCs proliferation, DCX migration and number of new integrated neurons). Our results showed positive but not significant correlations between the behavioral (positive slopes) and motor (negative slopes) outcomes and proliferation (Ki67-area of staining) and neuroblast migration in all the IL-1Ra treated groups and no correlation for the same outcomes and same neurogenesis markers in the placebo groups in young Wistar and Lean/Cp rats (data not shown). In contrast, for young Wistar rats, correlations between behavior score/new neurons at 28d (positive slope) and the motor score/new neurons at 28d (negative slope) were positive in the IL-1Ra treated group up to 14d and only significant in this group up to 7d post-ischemia, and no correlations in the Placebo young Wistar for these parameters were observed (24 h behavior score versus new neurons: R^2^ = 0.9454 and p = 0.02; 7d behavior score versus new neurons: R^2^ = 0.9 and p = 0.02; 24 h motor score versus new neurons: R^2^ = 0.92 and p = 0.04; 7d motor score versus new neurons: R^2^ = 0.95 and p = 0.04).

## 4 Discussion

Our findings demonstrate that subcutaneous administration of IL-1Ra is neuroprotective in young and aged animals with multiple risk factors for stroke and increases post-stroke neurogenesis.

It has previously been observed that delayed administration of IL-1Ra exerts neuroprotective effects at acute time points following experimental ischemia (Pradillo et al., 2012, Greenhalgh et al., 2010). Here we extend these findings to show that the early beneficial effects of IL-1Ra persist for at least 7 days in aged/co-morbid animals and for 28 days in young/healthy animals. It should be noted that there is significant infarct resorption over time, making it difficult to directly compare infarct volumes across the different timepoints. Delayed administration of IL-1Ra resulted in a reduced infarct volume, a preservation of BBB integrity and improvements in some functional outcomes.

A meta-analysis of pre-clinical research in cerebral ischemia showed in 2009 the potential benefit of IL-1Ra (Banwell et al., 2009) and in a recent cross-lab study performed across Europe, neuroprotective effects of IL-1Ra in experimental stroke were confirmed (Maysami et al., 2015). Furthermore, IL-1Ra showed some beneficial effects in a small phase II clinical study in acute stroke patients (Emsley et al., 2005). Our study confirms the protective nature of IL-1Ra in co-morbid animals at delayed time points, providing yet further validation of IL-1Ra as a highly promising therapy for ischemic stroke.

Obesity is commonly associated with increased ischemic damage due to raised levels of pro-inflammatory cytokines and proteases (Sola et al., 2009, McColl et al., 2010). Our data show that although 13-month-old corpulent rats had a plethora of stroke associated co-morbidities, infarct volumes were of a similar size to aged leans, suggesting that the extent of ischemic damage was close to maximal and that no further increase was possible. Conversely, younger rats were more resistant to the distal MCAO and occlusion time, showing only very small infarcts which necessitated a modification to the surgery protocol (tandem occlusion of the MCA and CCA) to attain similar infarcts across all strains for the purpose of examining neurogenesis. This suggests that age is the primary variable that increases the brain susceptibility to infarction following an ischemic stroke. However, despite reaching maximal levels of infarction, tissue is still salvageable under these circumstances if IL-1Ra is administered within a therapeutic window.

Edema often accompanies brain injury and is acknowledged as a critical component in the pathology of cerebral ischemia. The model of ischemia employed might explain the lack of reduction in edema seen here following IL-1Ra administration. In this model, a craniectomy is required to allow direct access and manipulation of the MCA, a procedure that alters intracranial pressure and thus the volume of edema (Doerfler et al., 1996, Altintas et al., 2015). Previous studies from our lab have shown significant improvements in edema in rats treated with IL-1Ra who have undergone transient MCAO using the filament model, a model that does not involve craniectomy (Girard et al., 2014, Greenhalgh et al., 2010).

To demonstrate that IL-1Ra not only reduces the infarct size but also improves the functional outcome, we performed in young Wistar and in aged/comorbid animals two general tests to analyze motor and behavioral deficits, and the cylinder test to determine forepaw asymmetry when the animal rears. Although with the first two tests we have been able to see an improvement in function with IL-1Ra treatment up to 14d in young animals, modest results were obtained with the cylinder test, only observing some improvements in rearing in young Wistar and aged Lean cohorts. One possible reason for this lack of effect is that the stroke model used here affects only the sensorimotor cortex and produces smaller infarct volumes when compared to other models (i.e. the filament model). Despite the modest results obtained in the cylinder test, our other results demonstrate that IL-1Ra improves the stroke outcome at longer time points (using the motor and behavioral scales) and that these improvements in function correlate with some neurogenesis markers. Beyond the acute phase of ischemic stroke, gross motor impairments generally resolve rapidly. It is therefore important to implement fine, long term motor testing to fully examine differences in genuine recovery and functional compensation and the effects of drug therapies on discrete motor deficits, as we have previously done with IL-1Ra (Girard et al., 2014). As well as motor impairments stroke survivors can experience a number of other complications, including cognitive decline, fatigue and depression. Evaluation of the effects of IL-1Ra and other potential stroke treatments on these parameters would be worthwhile, though at present the levels of such deficits in experimental stroke models remain poorly characterized.

The capacity of the brain to reorganize and repair itself after stroke can be seen clinically, even in the case of aged individuals (Minger et al., 2007). Aging and inflammatory co-morbidities are generally associated with a decline in general cerebral function, however the extent to which neurogenesis is affected under such conditions is unclear. To explore effects of IL-1Ra on markers of neurogenesis following ischemia it was essential to ensure initial infarct volumes were consistent across placebo groups, since there is a direct correlation between progenitor cell proliferation and infarct size in rats and mice at 7 days recovery (Moraga et al., 2014, Abeysinghe et al., 2014). In this study we observe that proliferating NPCs and neuroblasts are preserved in aged animals with different pre-existing inflammatory conditions, demonstrating the brain’s potential to repair itself is still viable in aged, co-morbid strains. Furthermore, our results indicate that although the delayed administration of IL-1Ra (3 and 6 h from reperfusion onset) reduces infarct volume, it produces an increase on cellular proliferation and migration of immature neurons versus placebo counterparts in the SVZ following stroke in young and aged/co-morbid rats, suggesting that a reduced inflammation of the tissue fosters a more efficient repair of the damaged tissue. We also show that IL-1Ra increases the number of new integrated neurons in areas surrounding the infarct lesion in young animals compared to placebo groups a result that correlates with improvements in motor and behavioral sub-acute outcomes. The benefits of IL-1Ra are therefore not limited to inducing neuroprotection, but also favor and promote neurorepair mechanisms.

Previous studies have examined the beneficial effects of IL-1Ra on neurogenesis in young healthy animals in models of chronic stress and Alzheimer’s disease like pathology (Ben Menachem-Zidon et al., 2008, Ben-Menachem-Zidon et al., 2014). In agreement with this data, the detrimental actions of IL-1β on adult neurogenesis have also been observed in young mice with an IL-1β excisionally activated transgene which results in chronic expression of this cytokine (Wu et al., 2012). Despite all these promising studies, the authors conclude that further studies are required to fully elucidate the mechanisms through which IL-1Ra may be mediating its beneficial, neurogenic effects.

In conclusion, we demonstrate that IL-1Ra protects against ischemic brain injury and improves functional recovery in aged/co-morbid animals when administered peripherally at delayed time points. Furthermore, IL-1Ra has the potential to improve neurogenesis in these animals, therefore providing a means of enhancing recovery in patients with a raised inflammatory burden.

## Competing financial interest

N.J.R. was a nonexecutive director of AstraZeneca when this work was completed (no involvement in this work). All the authors have no competing financial interests.
