# Supplementary material for: Reparative effects of interleukin-1 receptor antagonist in young and aged/co-morbid rodents after cerebral ischemia
Source: Brain Behav Immun. 2017 Mar;61:117–26. doi: 10.1016/j.bbi.2016.11.013 (PMC5325120; doi:10.1016/j.bbi.2016.11.013)
Supplement: Supplementary Figs 1–5 [file mmc1.pdf]

8. Supplementary Data

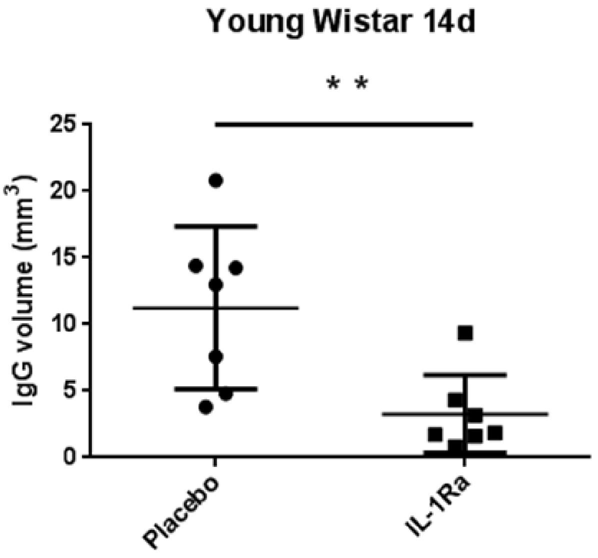

Supplemental figure 1: Effect of delayed administration of IL-1Ra/placebo on BBB damage measured at 14d after tMCAo in young Wistar (n=7). The volume of BBB damage was calculated on brain sections after IgG staining. Data are expressed as mean±SD. ***P*<0.01, Student´s t-test.

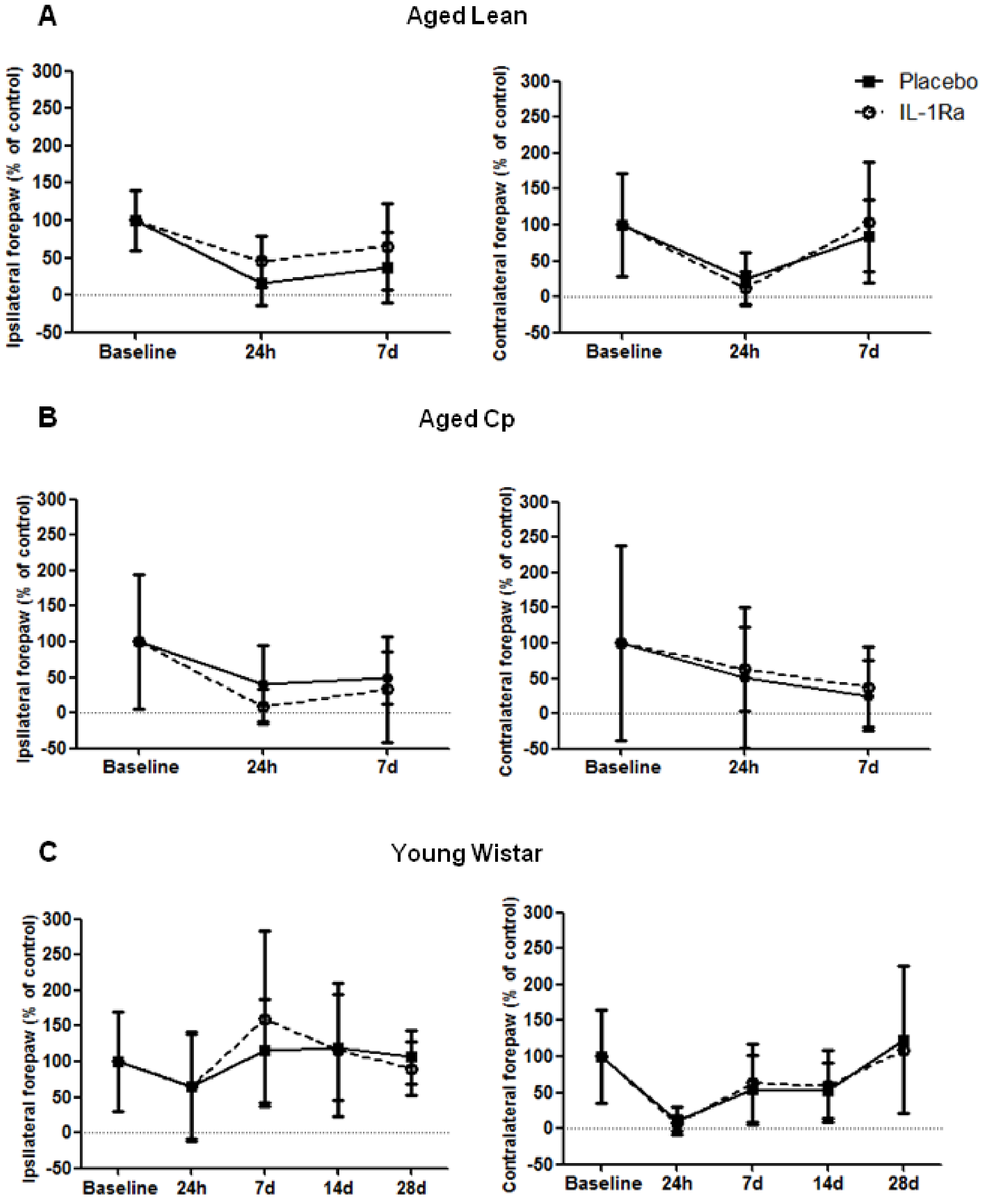

Supplemental figure 2: Effects of delayed administration of IL-1Ra/placebo (3 and 6h of reperfusion) on cylinder test in aged lean, aged Cp and young Wistar rats before and after experimental ischemia. The percentage of ipsilateral and contralateral forelimbs usage at 24h and 7d reperfusion was assessed in placebo and IL-1Ra treated aged lean

(**A**; n=10), aged corpulent (**B**; n=9) and young Wistar animals up to 28d after stroke (**C**; n=8) versus baseline. Data are presented as median and interquartile range.

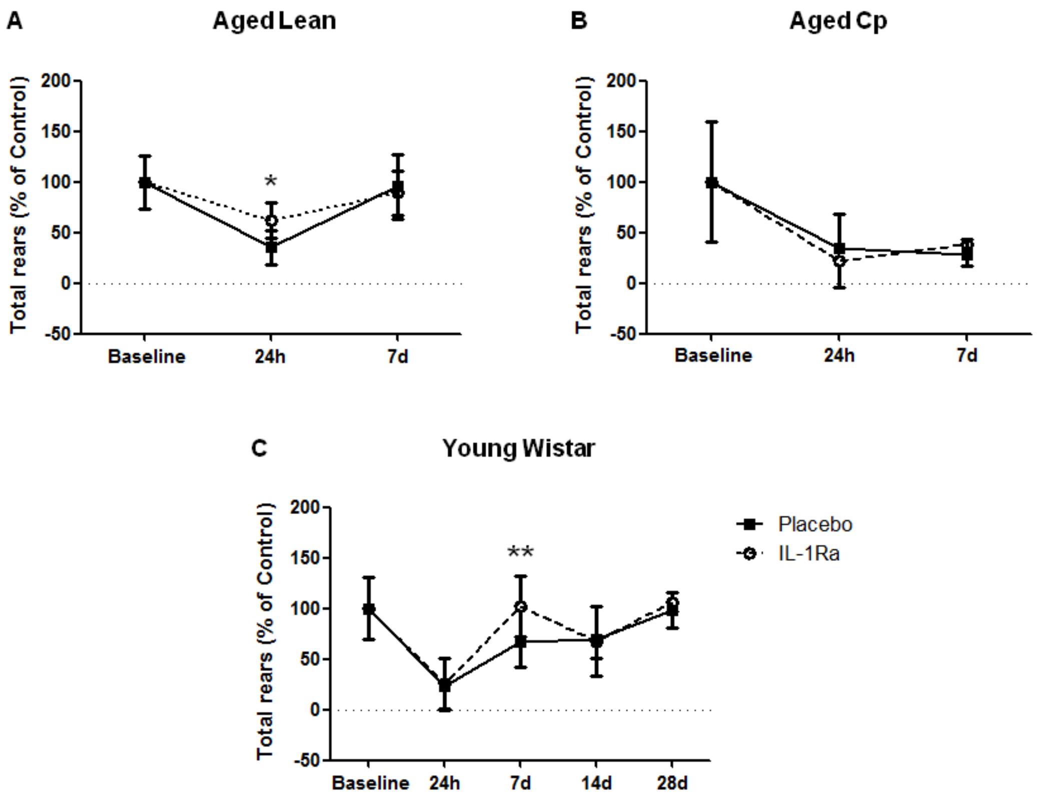

Supplemental figure 3: Effects of delayed administration of IL-1Ra/placebo (3 and 6h of reperfusion) on cylinder test: total rears**.** The percentage of total rears at baseline, 24h and 7d reperfusion was assessed in placebo and IL-1Ra treated aged lean (**A**; n=10), aged corpulent (**B**; n=9) and young Wistar animals (**C**; n=8) versus baseline. Data are presented as median and interquartile range. *P<0.05; **P<0.01, Mann-Whitney U test with Bonferroni correction.

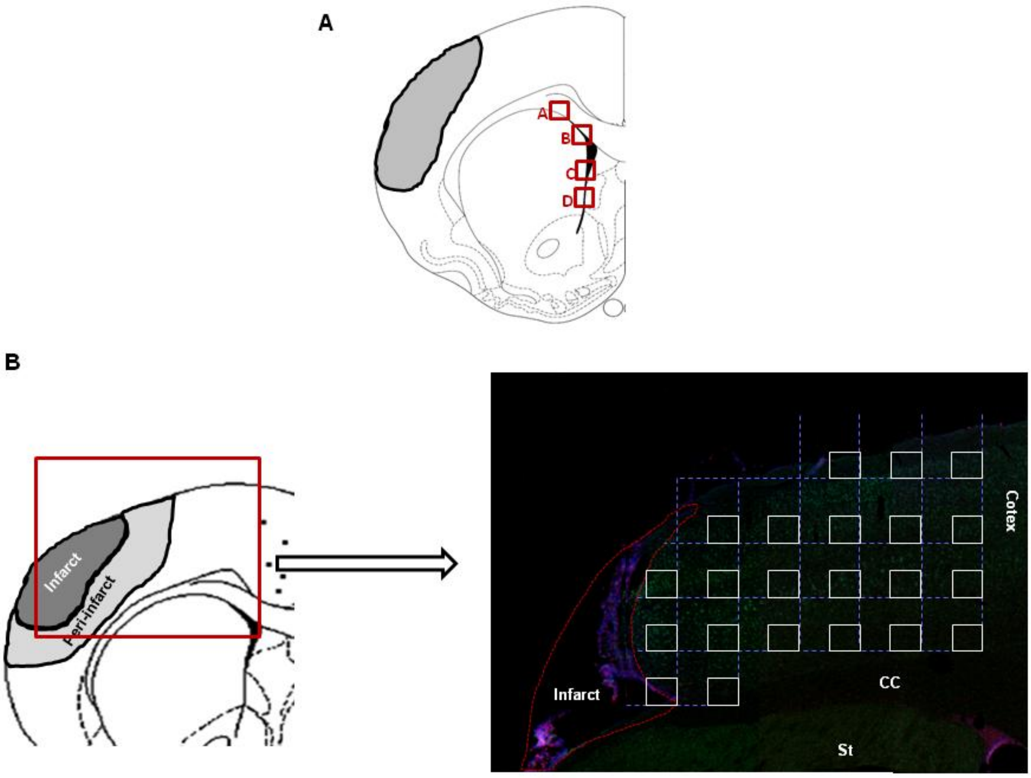

Supplemental figure 4: A: Image of a brain section showing the location of the different SVZ areas analyzed to determine the effect of IL-1Ra on NPCs proliferation at 7 days after stroke. The distance between image A and B was 200µm; distance between B-C and C-D was 400µm. **B:** Left, brain template showing the infarct and peri-infarct; right: representative image of a brain section stained for NeuN-BrdU-c-Fos showing different cortical regions of analysis. The new integrated neurons were found in the peri-farct region.

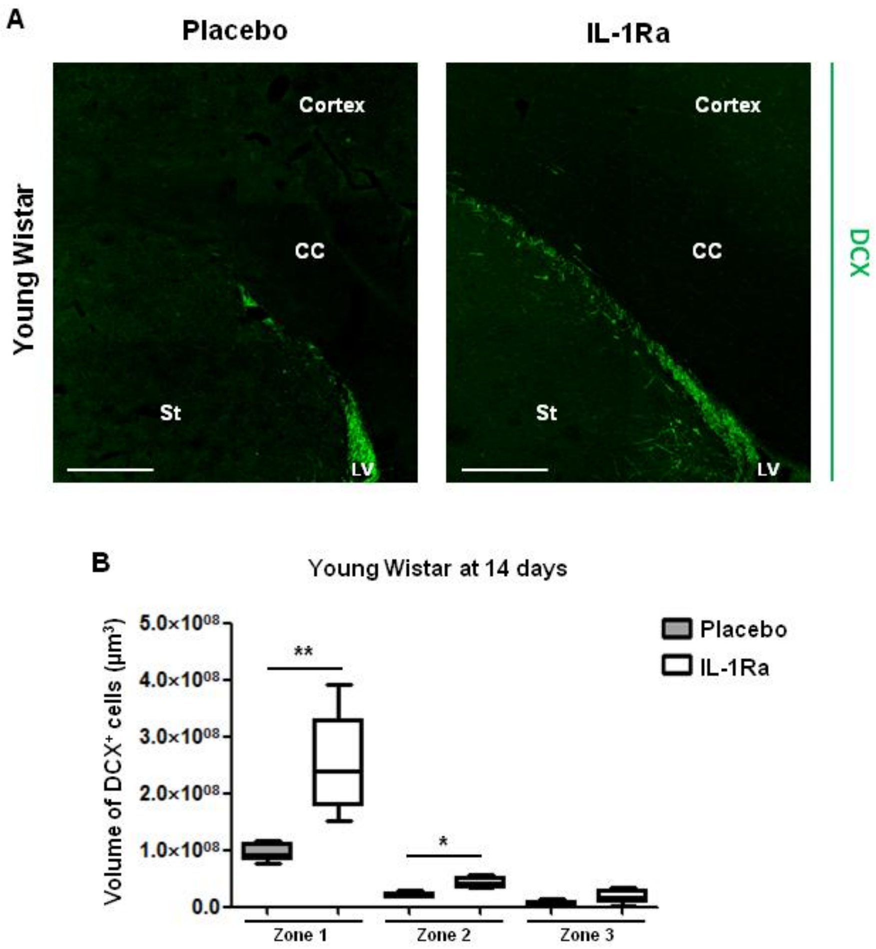

Supplemental figure 5: Effect of delayed administration of IL-1Ra on neuroblasts migration in young Wistar rats at 14d after transient cerebral ischemia. **A:** representative images of the different experimental groups; **B:** number of neuroblasts (DCX^+ cells) in 3 migratory zones (Zone1, Zone2, Zone3) at 14d after tMCAo in placebo or IL-1Ra treated young Wistar rats (n=8). Data are expressed as mean±SD. **P*<0.05; ***P*<0.01, Student´s t-test. Scale bar: 400µm.
